# Selective Impairment of T_H_17-Differentiation and Protection against Autoimmune Arthritis after Overexpression of BCL2A1 in T Lymphocytes

**DOI:** 10.1371/journal.pone.0159714

**Published:** 2016-07-19

**Authors:** Marcos Iglesias, Juan Jesús Augustin, Pilar Alvarez, Inés Santiuste, Jorge Postigo, Jesús Merino, Ramón Merino

**Affiliations:** 1 Departamento de Biología Molecular-IDIVAL Universidad de Cantabria, Santander, Spain; 2 Instituto de Biomedicina y Biotecnología de Cantabria, Consejo Superior de Investigaciones Científicas-Universidad de Cantabria, Santander, Spain; INSERM-Université Paris-Sud, FRANCE

## Abstract

The inhibition of apoptotic cell death in T cells through the dysregulated expression of BCL2 family members has been associated with the protection against the development of different autoimmune diseases. However, multiple mechanisms were proposed to be responsible for such protective effect. The purpose of this study was to explore the effect of the T-cell overexpression of BCL2A1, an anti-apoptotic BCL2 family member without an effect on cell cycle progression, in the development of collagen-induced arthritis. Our results demonstrated an attenuated development of arthritis in these transgenic mice. The protective effect was unrelated to the suppressive activity of regulatory T cells but it was associated with a defective activation of p38 mitogen-activated protein kinase in CD4^+^ cells after in vitro TCR stimulation. In addition, the in vitro and in vivo T_H_17 differentiation were impaired in BCL2A1 transgenic mice. Taken together, we demonstrated here a previously unknown role for BCL2A1 controlling the activation of CD4^+^ cells and their differentiation into pathogenic proinflammatory T_H_17 cells and identified BCL2A1 as a potential target in the control of autoimmune/inflammatory diseases.

## Introduction

The inhibition of cell death in lymphocytes has been repeatedly linked with the development of systemic autoimmune diseases. Thus, mice and humans with mutations in *fas/fasL*, transgenic (Tg) mice overexpressing human BCL2 (hBCL2) in B lymphocytes or mice with a targeted disruption of *BIM*, a pro-apoptotic BCL2 relative, develop an autoimmune syndrome resembling systemic lupus erythematosus (SLE) in association or not with lymphoproliferation [1–5]. Disease development in these situations is the consequence of the defective elimination of potentially harmful T and/or B cell clones either in primary lymphoid organs during development or in secondary lymphoid organs during or after lymphocyte activation [6–9].

In view of the above mentioned studies, a surprising observation was the protection against the development of autoimmune encephalomyelitis and diabetes in young BIM-deficient mice [10]. Similarly, the induction of graft-versus-host disease (GVHD) was impaired in these mutant mice [11]. In both studies, the protective effect was associated with a defective T-cell activation, that was manifested by a reduced activation of phospholipase C (PLC)γ1 [11] or by the inhibition of BCL2 interaction with inositol triphosphate receptor resulting in an impaired activation of nuclear factor of activated T-cells (NFAT), but not of mitogen-activated protein kinases (MAPK) or nuclear factor-kappa B (NF-κB) [10]. We also observed a protection against the development of collagen-induced arthritis (CIA) in mice overexpressing hBCL2 in T cells [12]. However, in these hBCL2 Tg mice the protection was mediated by regulatory T cells (Tregs) that showed an enhanced differentiation potential as well as an increased suppressive activity. Both phenomena were unrelated to the anti-apoptotic activity of BCL2, but dependent on its capacity to induce the T-cell expression of the cell cycle inhibitor p27^kip1^, that in turn, augmented the strength of TGFβ-signalling in these cells [12]. Other authors demonstrated that BCLX_L_ also promoted the development of Tregs, which ameliorate SLE following treatment with the hCDR1 tolerogenic peptide [13].

In this complex scenario, it seems that the consequences of inhibiting lymphocyte apoptosis, in terms of autoimmune disease development, may be determined by the lymphoid population in which the apoptotic program is disturbed, the age of the animal and/or the cell death regulator involved in the process. To further explore this problem, we study here the effects in the development of autoimmunity of the T-cell overexpression of BCL2A1 (also termed A1 or Bfl-1), another prosurvival member of the BCL2 family that together with MCL1 belongs to a different phylogenetic group than BCL2 and BCLX_L_ [6, 14]. Unlike BCL2 and BCLX_L_, BCL2A1 does not retard the cell cycle progression of T cells and does not affect cellular proliferation [15, 16]. Also, while the hydrophobic region at the C-terminal end of BCL2 and BCLX_L_ targets them to cellular membranes [17, 18], BCL2A1 can be found at different locations including mitochondria and cytoplasm [19]. Our results demonstrate that BCL2A1 overexpression in T cells protects mice against the development of CIA in association with a defective T_H_17 differentiation and p38 MAPK activation.

## Material and methods

### Ethics Statement

All studies with live animals were approved by the Universidad de Cantabria Institutional Laboratory Animal Care and Use Committee (refs 2014/12 and PI-02-15), carried out in accordance with the Declaration of Helsinki and the European Communities Council Directive (86/609/EEC) and all efforts were made to minimize suffering.

### Mice

C57BL/6 (B6) and DBA/1 mice were obtained from Harlan Ibérica (Barcelona, Spain). C3H/HeN-*lck-hBCL2* Tg mice [20] overexpressing hBCL2 selectively in T cells (BCL2-TgT) were obtained from the Jackson Laboratories (Bar Harbor, ME). The *Lck*.*hBCL2* transgene was transferred to B6 mice by backcross procedures as described previously [12]. B6-BCL2A1 Tg mice overexpressing BCL2A1a in T cells (B6-BCL2A1-TgT) have been described previously [16]. F1 hybrids between DBA/1 and B6 non-Tg (F1 non-Tg), B6-BCL2A1-TgT (F1-BCL2A1-TgT) or B6-BCL2-TgT (F1-BCL2-TgT) mice were bred in our animal facilities. B6-IL-17A-IRES-eGFP reporter mice (B6-IL-17/GFP) [21] were backcrossed with B6-BCL2A1-TgT mice in our animal facilities. Genotyping of mice was performed by PCR of genomic tail DNA.

### Induction of CIA, treatments and immunizations

Ten weeks old F1-BCL2-TgT, F1-BCL2A1-TgT and control littermate F1 non-Tg females were immunized at the base of the tail with 150 μg of bovine collagen type II (col II; MD Bioproducts, Zürich, Switzerland) emulsified with CFA containing 4 mg/ml of *Mycobacterium tuberculosis* (MD Bioproducts). The clinical and radiological evaluation of arthritis was performed, as described previously [12, 22]. Mice were killed 8 weeks after immunization and the hind paws were fixed in 10% phosphate-buffered formaldehyde solution and decalcified in Parengy’s decalcification solution overnight. The tissue was next embedded in paraffin. Sections (5 μm) were stained with hematoxylin and eosin.

For in vivo CD4^+^CD25^+^ Treg depletion, mice were treated ip with 0.5 mg/week of anti-CD25 mAb (clone PC61) from day 15 after col II immunization up to the end of the experiment. The efficiency of the treatment was evaluated by flow cytometry. Serum levels of IgG1 and IgG2a anti-col II antibodies were measured by ELISA 3 weeks after immunization. Briefly, microtiter plates (Maxisorp Nunc-immuno plates, ThermoFisher Scentific, Waltham, MA) were coated with col II (4 μg/ml) and the assay was developed with alkaline phosphatase-conjugated rat anti-mouse IgG1 or IgG2a (BD Biosciences, Franklin Lakes, NJ). Results were expressed in U/ml in reference to a standard curve obtained from a serum pool from col II-CFA immunized DBA/1 mice.

Mice were immunized with 400 μg of heat-aggregated human gammaglobulin (AHGG; Baxter S.L., Valencia, Spain) mixed with 1 mg of aluminum hydroxide (alum). Serum levels of IgG1 and IgG2a anti-HGG Ab were measured by ELISA and expressed in U/ml, as described [23].

### Gene expression analyses

The expression of mRNAs encoding for arthritogenic IL-1β, TNFα, IL-6 and IL-17A cytokines was explored in the paws before and 8 weeks after col II immunization by quantitative real time RT-PCR. Total RNA was obtained by TRIzol extraction (Invitrogen, ThermoFisher Scentific). One μg of the isolated RNA was used for cDNA synthesis with a RT-PCR kit (Amersham Pharmacia Biotech, Piscataway, NJ), according to the manufacturer instructions. Quantitative real time PCR (RT-qPCR) was performed on a StepOne Plus real time PCR instrument (Applied Biosystems, ThermoFisher Scentific) using specific TaqMan expression assays and universal PCR Master Mix (Applied Biosystems, ThermoFisher Scentific). Results (in triplicate) were normalized to *GAPDH* expression and measured in parallel in each sample.

### Cell cultures

Naïve CD4^+^CD25^-^CD62L^+^CD44^-^ cells from the different mouse strains were purified (more than 99% purity in all cases) by sorting on a FACSaria (BD Biosciences). For the differentiation cultures, 5x10^5^ naïve CD4^+^ cells were stimulated during 5 days with plastic-bound anti-CD3 (1 μg/well) and anti-CD28 (0.5 μg/well) mAbs (anti-CD3/CD28) under different polarizing conditions, as described previously [24]. The percentages of CD4^+^IFNγ^+^ (T_H_1), CD4^+^GATA-3^+^ (T_H_2), CD4^+^FoxP3^+^ (Treg) and CD4^+^IL-17^+^ (T_H_17) cells at the end of the culture period were evaluated by flow cytometry using commercially labeled antibodies (Biolegend, London, United Kingdom, and e-Bioscience Inc, San Diego, CA). CD4^+^ proliferation was measured after stimulation of cells with plastic-bound anti-CD3/CD28 during 3 days. Cultures were pulsed with 1 μCi of ^3^H-methyl-thymidine (^3^H-TdR) for the final 6 h of culture, harvested and counted. The kinetics of CD25 and CD69 induction in the stimulated cells were evaluated by flow cytometry.

### Apoptosis studies

Peripheral CD4^+^ cells were purified (97% purity) from the lymph nodes of the different groups of mice by magnetic beads and MACS (Miltenyi Biotec, Madrid, Spain). Thymocytes and lymph node CD4^+^ cells were cultured at 37°C in DMEM supplemented with 2 mM L-glutamine, 10^−5^ M 2-mercaptoethanol, and 10% heat-inactivated FCS (GE Healthcare Life Sciences, Logan, UT) and stimulated or not with plastic-bound anti-CD3/CD28 mAbs. The viability of thymocytes was explored at different time points by trypan blue exclusion. The presence of lymph node CD4^+^ cells undergoing apoptosis at the indicated time points was assessed by annexinV staining (BD Biosciences). In some experiments mice were treated ip with 2 mg of dexamethasone sodium phosphate (American Regent Laboratories, Shirley, NY) and the number of CD4^+^CD8^+^ thymocytes was analyzed 48 h later by flow cytometry.

### Western blotting

The expression levels of either dephosphorylated and phosphorylated NFATc2 (dNFAT and pNFAT) in comparison to those of β-actin and of phosphorylated IκB (pIκB), ERK-1/2 (pERK) and p38 (pp38) in comparison to those of total IκB, ERK-2 and p38, respectively, were detected by Western blotting in cell lysates from purified CD4^+^ cells at different time points after in vitro stimulation with anti-CD3/CD28 mAbs, using specific antibodies (Santa Cruz Biotechnology, Heidelberg, Germany). The relative band intensities of these proteins in comparison to their respective controls were determined by densitometry using ImageJ software.

### Flow Cytometry

The percentages of T_H_17 cells in the spleen of F1 non-Tg and F1-BCL2A1-TgT mice before and 21 days after col-II immunization and in the lamina-propria (LP) of non-immunized non-Tg-IL-17/GFP and BCL2A1-IL-17/GFP mice were determined by flow cytometry using commercially labeled antibodies (Biolegend and eBioscience). Intracellular cytokine staining was performed using an intracellular staining kit (BD Biosciences), as described previously [12]. Cells were analyzed in a FACSCanto II flow cytometer using FACSDiva software (BD Biosciences).

### Statistical analysis

Differences between 2 groups were analyzed by a 2-tailed Student’s *t* or 2-sample Mann-Whitney *U* tests. Probability values <0.05 were considered significant.

## Results

### Overexpression of BCL2A1 in T cells inhibits the development of CIA

**Fig 1:**
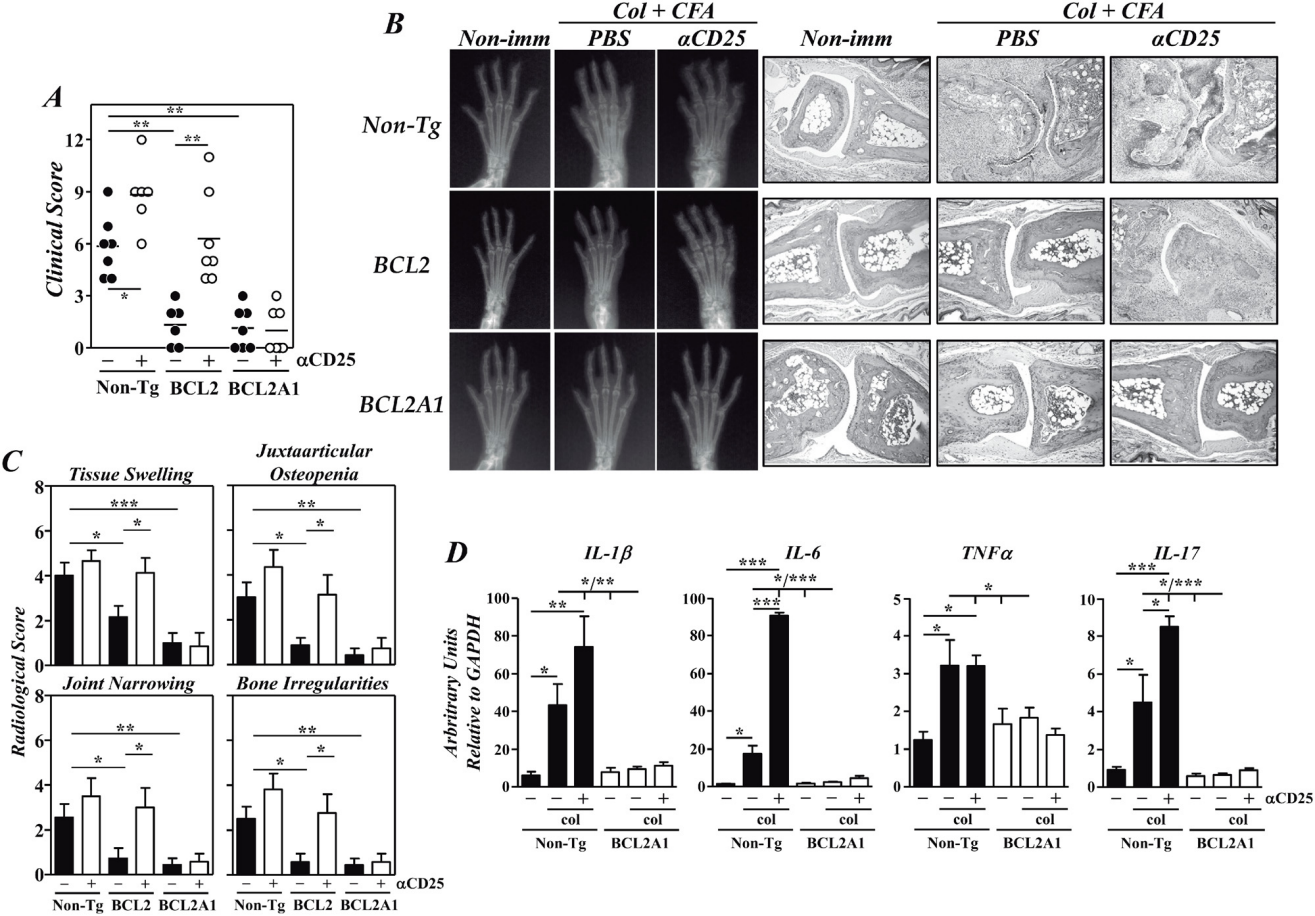
Protection against CIA in BCL2A1 Tg mice. Ten weeks-old F1-BCL2-TgT, F1-BCL2A1-TgT and control F1 non-Tg littermates, depleted or not of CD4^+^CD25^+^ Tregs after treatment with an anti-CD25 mAb, were immunized with col-II-CFA. (A) Clinical severity of arthritis in individual mice 8 weeks after col-II immunization. Bars represent mean values. (B) Representative radiological and histological (x10) images in the different experimental groups 8 weeks after immunization. (C) Radiological scores of different radiological signs associated with disease severity expressed as the mean ± SD. Results from A to C are representative of five independent experiments. (D) Expression of mRNAs encoding for arthritogenic cytokines in the paws of non-immunized and untreated or anti-CD25 treated col-II-CFA immunized F1 non-Tg mice (closed bars) and F1-BCL2A1-TgT (open bars) 8 weeks after immunization analyzed by RT-qPCR. Results are expressed as the mean ± SD fold change (n = 6–7 mice/group) of each cytokine relative to GAPDH expression measured in parallel in each sample. Statistic differences are indicated as follow: *p<0.05, **p<0.01, *** p<0.001. When not indicated, differences did not reach statistical signification.

We first explored whether the T-cell overexpression of BCL2A1 changed the development of CIA. As previously described [12], F1-BCL2-TgT and non-Tg mice developed a mild or a severe CIA, respectively (Fig 1A). Interestingly, F1-BCL2A1-TgT mice also developed an attenuated CIA in comparison to F1 non-Tg controls (Fig 1A). The severity of different radiological signs associated with disease activity was clearly reduced in the joints of immunized F1-BCL2A1-TgT and F1-BCL2-TgT mice (Fig 1B and 1C). These radiological findings were confirmed by histology, showing the presence of cartilage and bone destruction, synovitis and pannus formation in the joints of F1 non-Tg mice, but not of F1-BCL2A1-TgT and F1-BCL2-TgT mice (Fig 1B). A significant increase in the levels of mRNAs encoding for arthritogenic IL-1β, TNFα, IL-6 and IL-17 cytokines was observed in the paws of F1 non-Tg mice, but not in those of F1-BCL2A1-TgT mice, 8 weeks after immunization with col-II (Fig 1D).

**Fig 2:**
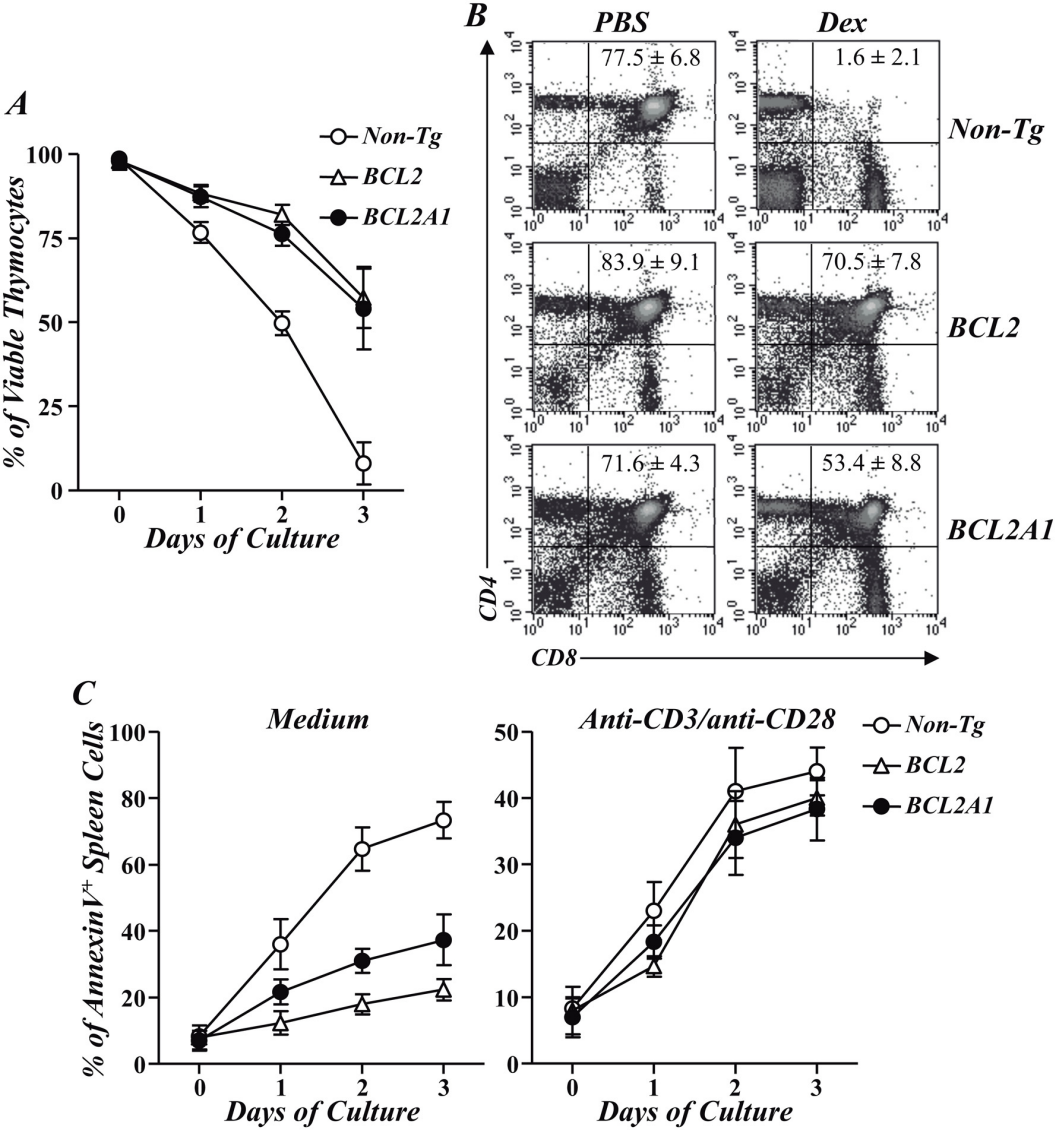
Anti-apoptotic effects of T-cell overexpression of BCL2 and BCL2A1. (A) Overexpression of BCL2 or BCL2A1 increases thymocyte viability in vitro. Thymocytes from the different experimental groups were cultured in DMEM supplemented with 10% FCS. The percentage of viable thymocytes was assessed from day 0 to 3 by trypan blue exclusion. Results represent the mean of triplicate cultures ± SD for three independent experiments. (B) Overexpression of BCL2 or BCL2A1 blocks thymocyte dexamethasone-induced cell death in vivo. Mice were injected ip with 2 mg of dexamethasone and compared with control mice injected with PBS. Representative flow cytometry dot plots of CD4^+^CD8^+^ thymocytes 48 h after treatment. Mean values ± SD of this cell population in each experimental group (4 mice/group) from one out 3 independent experiments are indicated. (C) In vitro apoptosis of purified lymph node CD4^+^ cells from BCL2-TgT, BCL2A1-TgT and non-Tg mice stimulated (right) or not (left) with anti-CD3/CD28 mAbs. The percentage of annexinV^+^ apoptotic cells was assessed from day 0 to 3 by flow cytometry. Results represent the mean of triplicate cultures ± SD for three independent experiments.

We have previously reported that Tregs were responsible for the protection against CIA in F1-BCL2-TgT mice [12]. In order to assess whether a similar mechanism operated in F1-BCL2A1-TgT mice, these animals were depleted in CD4^+^CD25^+^ Tregs with a cytotoxic anti-CD25 mAb. Unlike anti-CD25 treated F1-BCL2-TgT mice, the clinical, radiological and histological severity of CIA in F1-BCL2A1-TgT mice was not modified after this treatment (Fig 1A–1C), indicating that the protection was not mediated by Tregs. A significant exacerbation of CIA was also observed in anti-CD25 treated F1 non-Tg mice in association with an increased paw expression of IL-6 and IL-17 mRNAs but not of IL-1β and TNFα mRNAs (Fig 1D). The reason for the discrepancy in the mechanism of CIA protection between both strains of Tg mice was not explained by differences in the capability of BCL2A1 and BCL2 to inhibit several forms of T-cell death. In fact, thymocytes and purified lymph node CD4^+^ cells from BCL2A1-TgT and BCL2-Tg mice showed an improved in vitro survival after culture in medium supplemented with 10% FCS compared with cells from non-Tg controls (Fig 2A and 2C; p<0.005). Furthermore, double positive thymocytes were almost entirely eliminated in non-Tg controls 48 hrs after ip injection of 2 mg of dexamethasone, whereas 84% and 75% of this population remained viable in BCL2-Tg and BCL2A1-TgT mice, respectively (Fig 2B). However, the death of lymph node CD4^+^ cells induced after anti-CD3/CD28 activation was not affected by BCL2A1 or BCL2 overexpression (Fig 2C; p>0.1).

**Fig 3:**
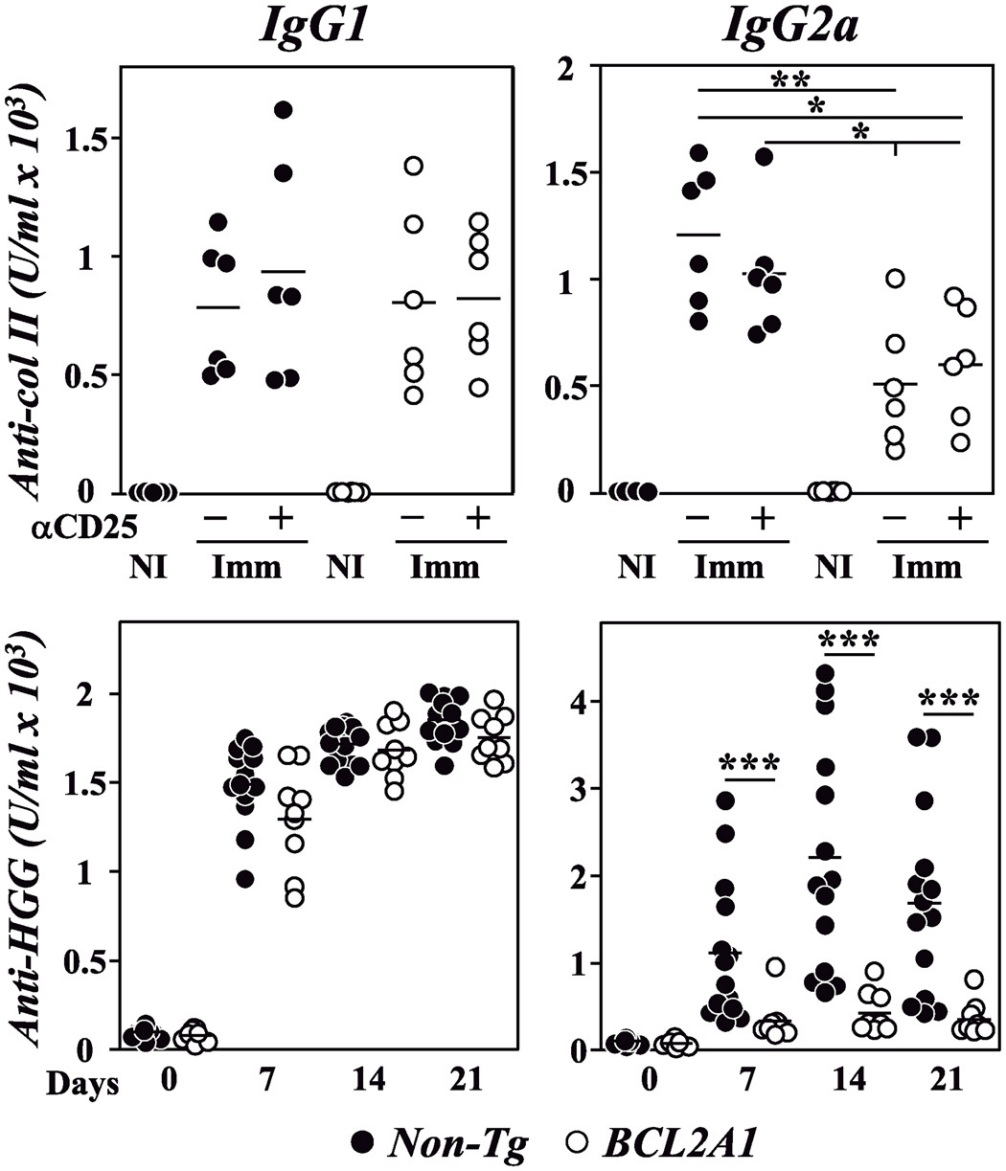
Effect of T-cell overexpression of BCL2A1 in humoral immune responses. BCL2A1-TgT and non-Tg mice were immunized with col II-CFA or AHGG-alum. Serum levels of IgG1 and IgG2a anti-col II (upper panels) and anti-HGG antibodies (lower panels) were determined by ELISA before and after immunization. Results of individual mice and mean values in one of two independent experiments are represented. Statistic differences are indicated as follow: *p<0.05, **p<0.01, *** p<0.001. When not indicated, differences did not reach statistical signification.

The intensity and quality of anti-col II humoral immune responses were next compared between F1 non-Tg and F1-BCL2A1-TgT mice, treated or not with anti-CD25 mAb, by analyzing the levels of circulating IgG1 and IgG2a anti-col II antibodies. No differences in IgG1 anti-col II antibody responses were observed between the different experimental groups of immunized mice, independently of the treatment received (Fig 3). However, the circulating levels of IgG2a anti-col II antibodies were significantly decreased in F1-BCL2A1-TgT mice (Fig 3). To further confirm the effect of T-cell overexpression of BCL2A1 in IgG2a humoral immune responses, BCL2A1-TgT and non-Tg mice were immunized with the T-dependent antigen AHGG mixed with alum. Again, the levels of IgG2a anti-HGG antibodies, but not of IgG1 anti-HGG antibodies, were clearly reduced in BCL2A1-TgT mice in comparison to F1 non-Tg controls (Fig 3).

### Activation status of CD4^+^ cells in BCL2A1-TgT mice

**Fig 4:**
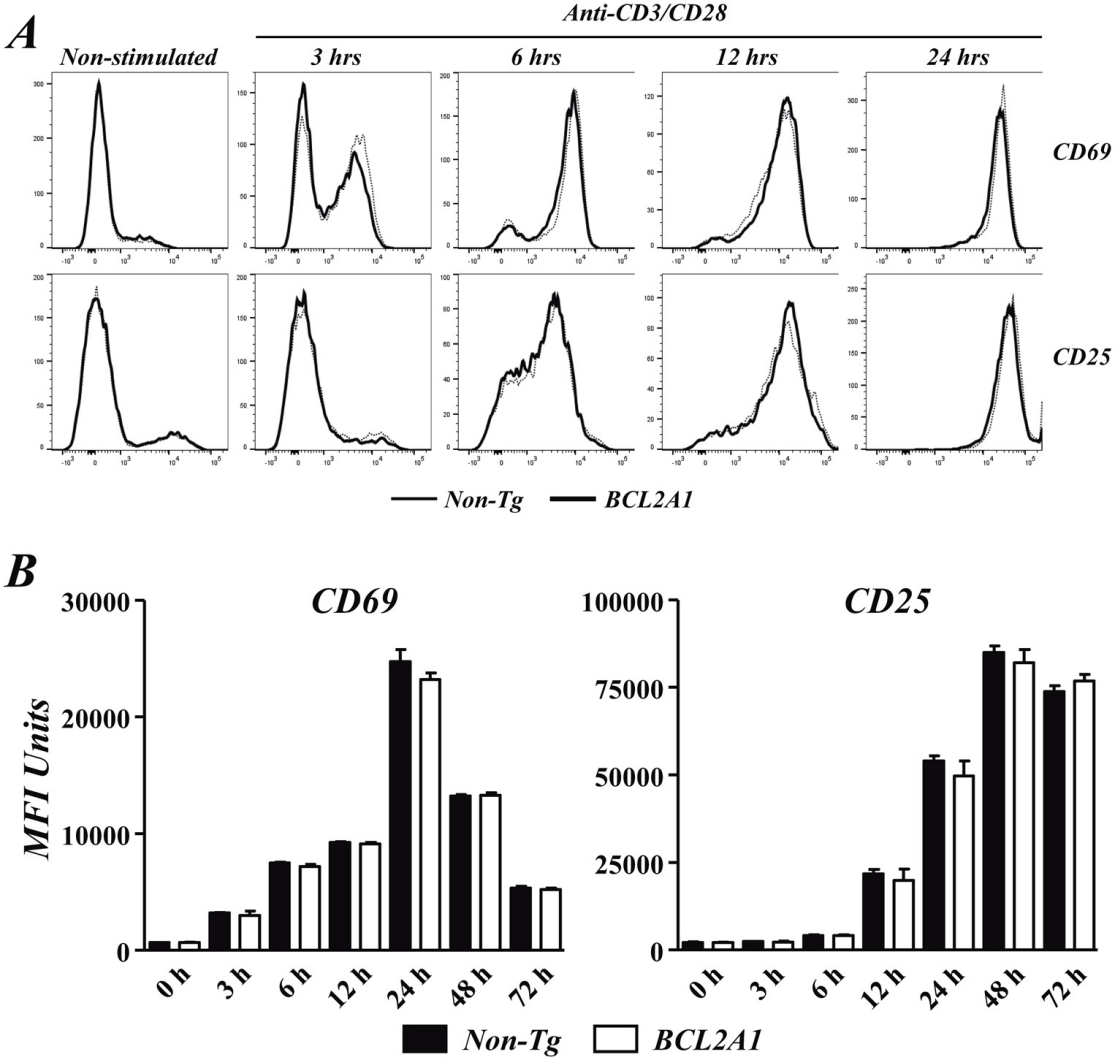
Effect of BCL2A1 overexpression in the kinetics CD69 and CD25 induction in activated CD4^+^ cells. Purified naïve CD4^+^ cells from BCL2A1-TgT and non-Tg mice were stimulated in vitro with anti-CD3/CD28 mAbs and the kinetics of CD69 and CD25 induction in these cells were explored by flow cytometry. (A) Representative overlapping histograms of CD69 and CD25 expression in CD4^+^ cells from non-Tg (dotted line) and BCL2A1-TgT (solid line) at different time points after activation. (B) Mean ± SD of MFI values of CD69 and CD25 expression in CD4^+^ cells from non-Tg (open bars) and BCL2A1-TgT (closed bars) at different time points after activation. Results are representative of four independent experiments. When not indicated, differences did not reach statistical signification.

**Fig 5:**
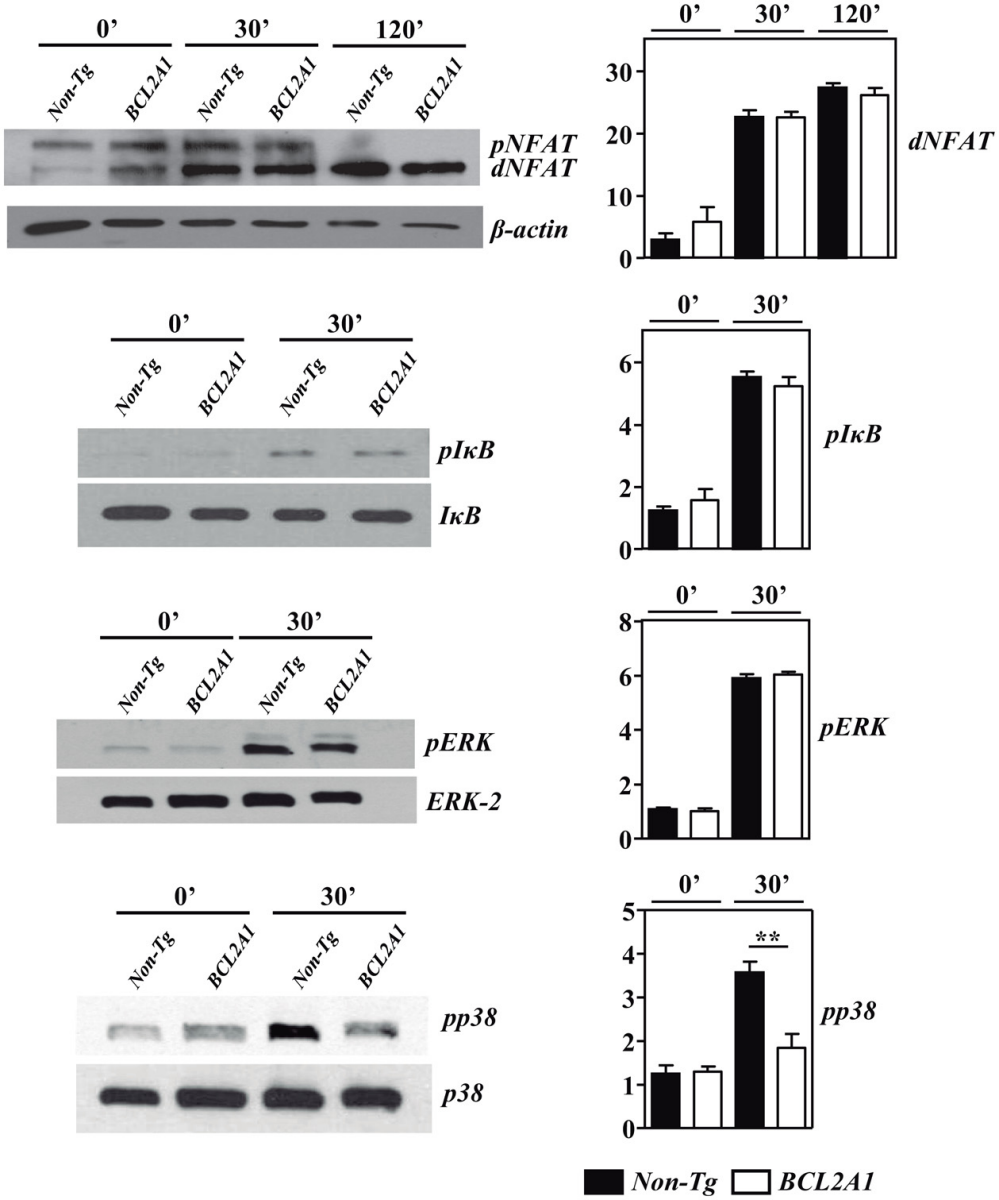
Defective p38 MAPK activation in CD4^+^ cells from BCL2A1 Tg mice. CD4^+^ cells from BCL2A1-TgT and non-Tg mice were stimulated in vitro with anti-CD3/CD28 mAbs. The expression of dephosphorylated and phosphorylated NFATc2 (dNFAT and pNFAT, respectively) in comparison to that of β-actin, of phosphorylated IκB (pIκB) in comparison to total IκB and of phosphorylated ERK (pERK) and p38 (pp38) MAPKs in comparison to total ERK and p38 in CD4^+^ cells from BCL2A1-TgT and non-Tg mice at different time points after activation was determined by western blot (left panels). Right panels show mean ± SD of the relative band intensities of these proteins in comparison to their respective controls of three-five independent experiments. Statistic differences are indicated as follow: **p<0.01. When not indicated, differences did not reach statistical signification.

Based on previous observations in young BIM-deficient mice [10, 11], the in vitro activation of CD4^+^ cells was compared between BCL2A1-TgT and non-Tg mice. No differences in the anti-CD3/CD28-induced proliferation of CD4^+^ cells were observed between both strains of mice (^3^H-TdR counts in non-stimulated CD4^+^ cells from non-Tg mice: 0.3 ± 0.1 x 10^3^; from BCL2A1-TgT mice: 0.5 ± 0.2 x 10^3^; in anti-CD3/CD28 stimulated CD4^+^ cells from non-Tg mice: 12.8 ± 3.2 x 10^3^; from BCL2A1-TgT mice: 11.7 ± 2.6 x 10^3^). In addition, the kinetics of CD69 or CD25 induction in CD4^+^ cells after their activation were also similar in BCL2A1-TgT and non-Tg mice (Fig 4). We next studied the TCR-induced activation of MAPK, NF-κB and NFAT pathways in CD4^+^ cells from BCL2A1-TgT and non-Tg mice. The activation-associated dephosphorylation of NFATc2 and phosphorylation of IκB and ERK MAPK were similar in CD4^+^ cells from both origins (Fig 5). However, a reduced phosphorylation of p38 MAPK was noticed in CD4^+^ cells from BCL2A1-TgT mice after anti-CD3/CD28 activation (Fig 5).

### Selective in vitro and in vivo impairment of T_H_17 cell differentiation in BCL2A1-TgT mice

**Fig 6:**
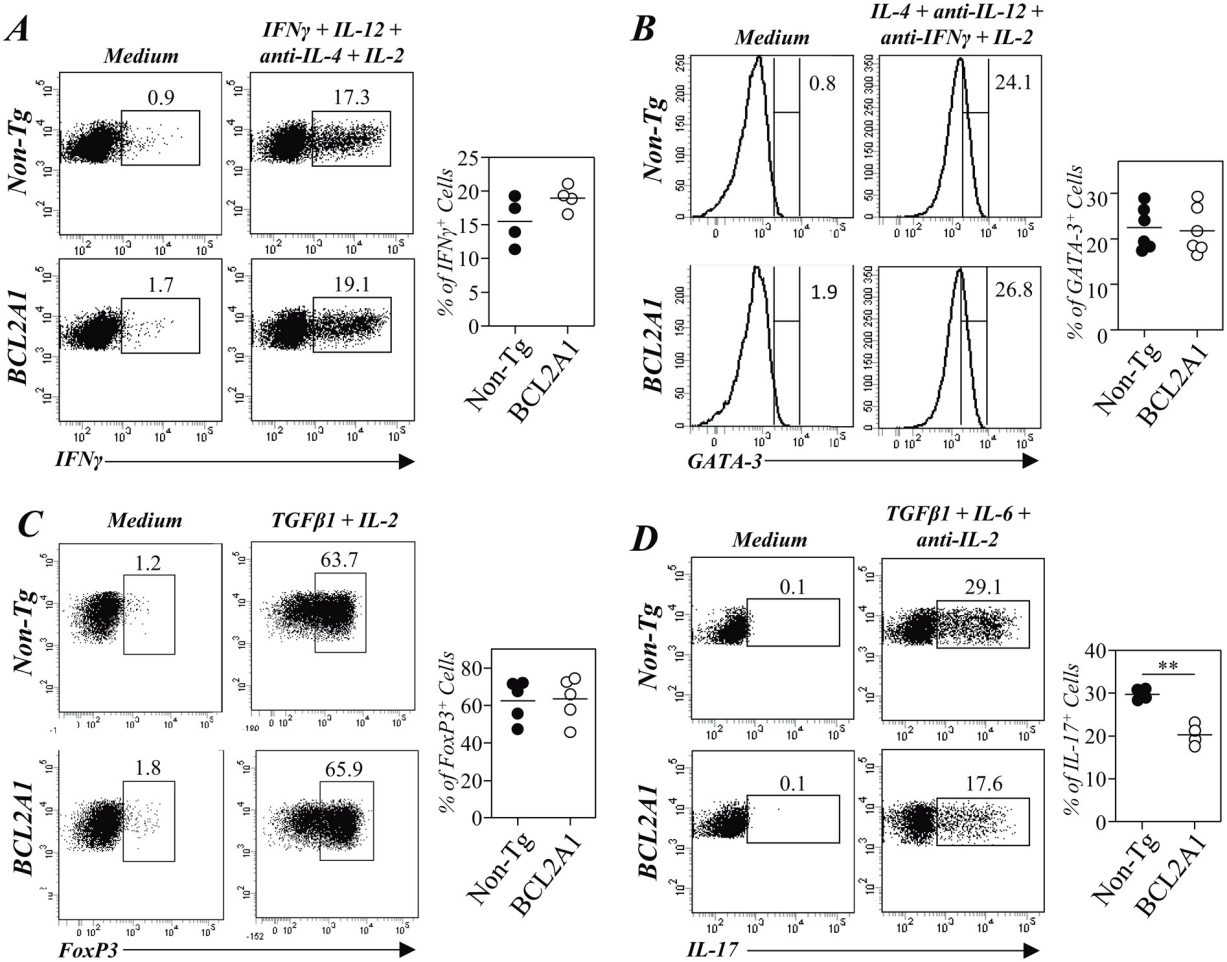
BCL2A1 selectively inhibits in vitro T_H_17 differentiation. Naïve CD4^+^ cells from BCL2A1-TgT and non-Tg mice were stimulated under T_H_1 (A), T_H_2 (B), Treg (C) or T_H_17 (C) polarization conditions. Representative flow cytometry dot plots or histograms and percentages of CD4^+^IFN^+^ (T_H_1; A), CD4^+^GATA-3^+^ (T_H_2; B) CD4^+^FoxP3^+^ (Treg; C) and CD4^+^IL-17^+^ (T_H_17; D) cells after 5 days of culture. Cultures under T_H_0 conditions are included for comparison. Results of multiple experiments are also plotted together. Mean values are indicated. Statistic differences are indicated as follow: **p<0.01. When not indicated, differences did not reach statistical signification.

We explored whether the CD4^+^ activation defects observed in BCL2A1-TgT mice were associated with changes in their in vitro functional differentiation capability. To this end, naïve CD4^+^ cells from B6 non-Tg and BCL2A1-TgT mice were activated in vitro with anti-CD3/CD28 antibodies during 5 days under different polarization conditions [24]. No differences in the in vitro T_H_1, T_H_2 and Treg differentiation were observed between CD4^+^ cells from B6 non-Tg and BCL2A1-TgT mice (Fig 6A–6C). However, the in vitro T_H_17 differentiation was significantly reduced in BCL2A1-TgT mice in comparison to non-Tg controls (Fig 6D).

**Fig 7:**
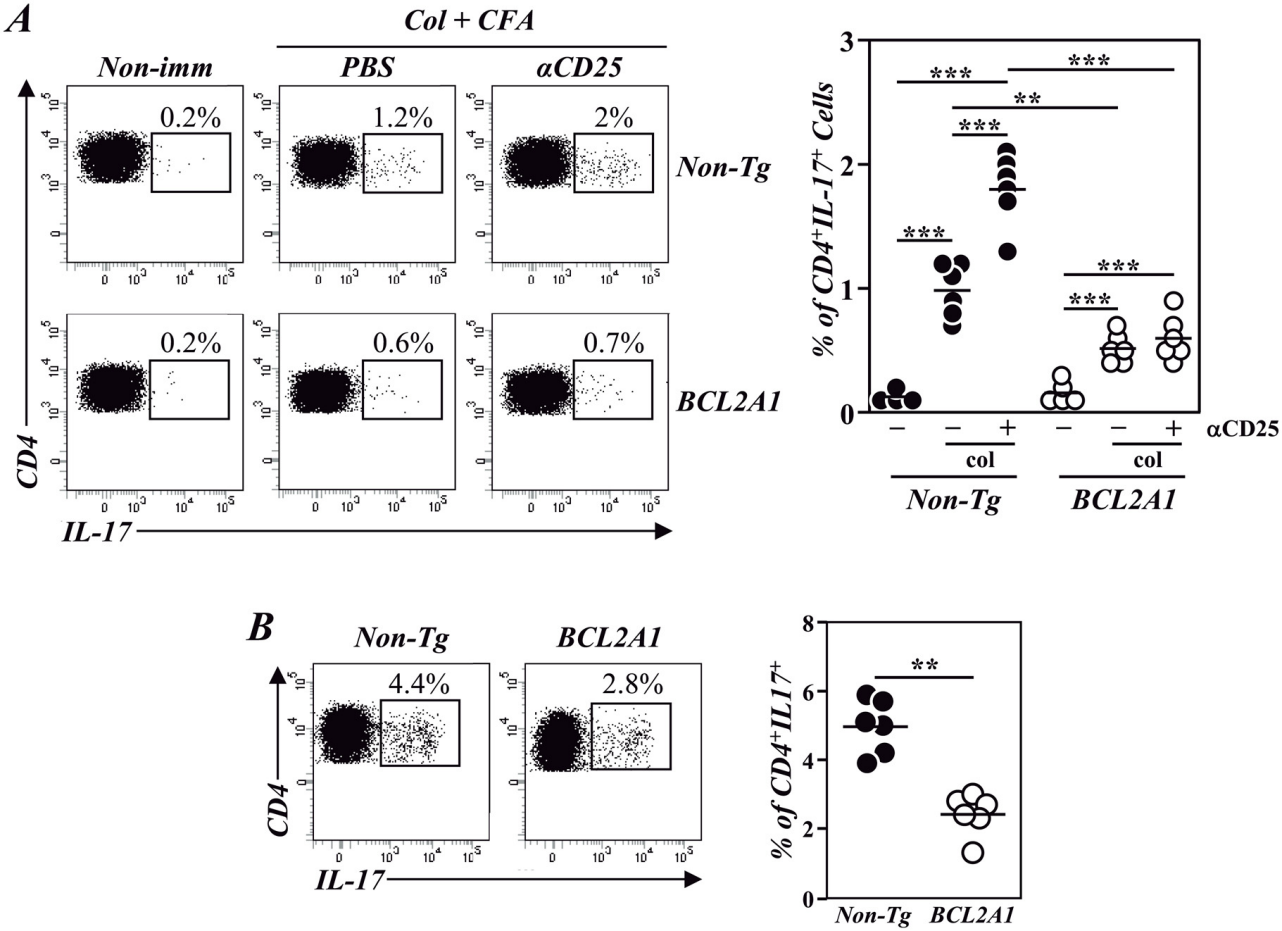
Effect of T-cell overexpression of BCL2A1 in T_H_17 induction under pro-inflammatory or homeostatic conditions. (A) Induction of T_H_17 cells during CIA and effect of anti-CD25 treatment. BCL2A1-TgT and non-Tg mice were immunized with col-II-CFA and treated or not with an anti-CD25 mAb. The percentages of T_H_17 in the spleen before and 3 weeks after immunization were determined by flow cytometry. (B) Percentages of TH17 cells in the LP of the colon of non-manipulated BCL2A1-TgT-IL-17/GFP and non-Tg-IL-17/GFP mice determined by flow cytometry. In A and B left panels show dot plots and percentages in one representative animal and right panels shows individual percentages of these cells in a group of mice from one of 3–4 independent experiments, respectively. Mean values are indicated. Statistic differences are indicated as follow: **p<0.01, ***p<0.001. When not indicated, differences did not reach statistical signification.

We finally evaluated the T_H_17 differentiation/expansion in vivo. First, the percentages of T_H_17 cells were compared in the spleen of F1 non-Tg and F1-BCL2A1-TgT before (steady state) and after induction of CIA, a well-established model of T_H_17-dependent autoimmune disease [25, 26]. T_H_17 cells were barely detected before immunization with col-II emulsified with CFA in the spleen of both strains of mice but were significantly increased in F1 non-Tg mice after immunization (Fig 7A). This increase was even higher after depletion of CD4^+^CD25^+^ Tregs (Fig 7A). In correlation with the in vitro differentiation studies, the increase in T_H_17 cells after col-II immunization was more limited in F1-BCL2A1-TgT mice and was unaffected after anti-CD25 depletion (Fig 7A).

Because T_H_17 cells are almost undetectable in the spleen of non-immunized mice (Fig 7A), we further compare the percentages of T_H_17 cells in a location with a high representation of this cell population under homeostatic conditions. This is the case of the LP of the colon of non-manipulated mice [21]. To facilitate the detection of T_H_17 cells by flow cytometry, that normally requires the in vitro activation of T cells in the presence of a Golgi protein transport inhibitor [12], these experiments have been performed in IL-17/GFP reporter mice in which T_H_17 cells express GFP without the necessity of an additional in vitro activation (21). In these mice, the percentages of T_H_17 cells in the LP of the colon are significantly lower in BCL2A1-TgT mice than in non-Tg controls (Fig 7B).

## Discussion

Natural or induced genetic mutations in cell death regulators or alterations in their expression pattern in immune cells have been associated with either the induction or the inhibition of autoimmunity [1–5, 10, 11]. To gain insights into the potential mechanisms by which the inhibition of lymphocyte apoptosis conferred disease protection, in the present study we explored the effects of T-cell overexpression of BCL2A1 in the development of CIA. Our results demonstrated that BCL2A1-TgT mice developed an attenuated disease in association with an impaired differentiation of T_H_17 cells and a defective activation of p38 MAPK signaling pathway.

From previous studies and our present observation it can be inferred that the effects of dysregulating cell death in the control of lymphoid homeostasis are determined by the apoptotic pathway involved, by the cellular context where such defects occurs and by the age of the animal. Thus, the inhibition of the extrinsic apoptotic pathway results in the induction of autoimmunity [2, 3]. In contrast, the effects of inhibiting the intrinsic cell death pathway by the altered expression of BCL2 family members are clearly cellular dependent. Whereas the inhibition of B-cell apoptosis, observed in old BIM deficient mice or after the B-cell overexpression of BCL2, causes SLE in predisposed animals [3–5], the dysregulation of this pathway in T cells blocks the development of autoimmune diseases in young BIM deficient mice or in BCL2- or BCLX-TgT mice [10–13]. Our present study showing that BCL2A1-TgT mice are protected against the development of CIA further support these observations. Although the consequences of BCL2A1 overexpression in B cells are still unknown, it has been reported that its expression is upregulated after BCR signaling and in patients with SLE [27, 28].

It was reported in a murine model of proteoglycan-induced arthritis that disease severity inversely correlated with the extent of activation-induced cell death (AICD) [29, 30], an apoptotic process dependent on cell death receptor and extrinsic apoptotic pathways [7, 31]. However, it should be remarked that AICD induction in peripheral CD4^+^ cells was not affected by BCL2 or BCL2A1 overexpression that otherwise efficiently inhibited other forms of T-cell death. These observations suggested that the cell death inhibitory activities of BCL2 and BCLA1 were not responsible for the protection against CIA development observed in both strains of Tg mice. Instead, the present and previous studies were compatible with a model in which disease protection could be mechanistically linked with the role that each particular BCL2 family member might play in the regulation of CD4^+^ cell activation and/or functional differentiation. While the protection against autoimmune diseases in mice overexpressing BCL2 in T cells was secondary to the capacity of this anti-apoptotic molecule to control the expression of the cell cycle inhibitor p27^kip1^ that in turn, regulated the differentiation and activity of Tregs [12], the protection observed in young, but not old, BIM^-/-^ [5, 10, 11] and in BCL2A1 TgT mice was associated with defects in the activation of T cells. Moreover, BIM deficiency and BCL2A1 overexpression modulated CD4^+^ cell activation at different levels ([10, 11] and the present study).

It has been clearly demonstrated the crucial role played by T_H_17 cells in the pathogenesis of inflammatory/autoimmune diseases [25, 26]. The combination of genome-wide transcription factor occupancy studies with gene expression profiles in both purified T_H_17 populations and single T_H_17 cells allow the characterization of a genetic regulatory network accounting for T_H_17 differentiation [32, 33]. One interesting observation of these studies is that the expression of BCL2A1 appears downregulated during T_H_17 differentiation [32, 33]. In addition, all-trans retinoic acid or retinoic X receptor agonists, that inhibit T_H_17 differentiation [34–36], are potent transcriptional inducers of BCL2A1 expression [37–39]. However, the biological significance of such findings in terms of T_H_17 differentiation is unknown. Our present study describes for the first time a functional role for BCL2A1 in the differentiation of T_H_17 cells. Thus, the in vitro T_H_17 differentiation of activated CD4^+^ cells is severely reduced in BCL2A1-TgT mice as well as their in vivo induction under homeostatic or inflammatory condition in the intestinal LP of non-manipulated mice and in the spleen of animals during CIA induction, respectively. Moreover, a selective impairment of IgG2a humoral immune responses, which have been associated with T_H_17 cells [40, 41], is observed in these Tg mice. This last aspect may be particularly relevant in our study since B cells and antibodies are involved in the development of CIA in mice [42, 43]. Also, it has been demonstrated that the capacity of IgG autoantibodies to promote tissue damage is greatly influenced by the IgG subclass. By comparing the capacity to induce hemolytic anemia of several IgG isotype-switch variants of a pathogenic anti-red blood cell autoantibody, Fossati-Jimack et al have demonstrated in an elegant study that the IgG2a switch variant is about 20 times more pathogenic than the IgG1 variant [44], and that the distinct pathogenicity of these variants correlates with the different affinities of IgG2a and IgG1 antibodies for Fcγ receptors, promoting antibody-dependent cellular cytotoxicity [45], and with the higher capacity of IgG2a antibodies to activate the complement cascade [46]. Consistent with this, we propose here that through the inhibition of T_H_17-associated cytokine production and their effects in the control of cellular inflammatory responses or bone remodeling [26, 47], and through the qualitative modulation of humoral immune responses, the reduced T_H_17 differentiation can be responsible for the protection against CIA in BCL2A1 TgT mice.

Concerning the mechanism involved in the regulation of T_H_17 differentiation by BCL2A1, we demonstrate that the overexpression of this anti-apoptotic molecule affects the TCR-induced activation of p38 MAPK in CD4^+^ cells, but not of ERK MAPK or the NF-κB and NFAT signaling pathways. Interestingly, different reports have demonstrated the importance of p38 MAPK for the differentiation and function of T_H_17 cells both in humans and mice [48–50]. Although we have not clarified in our study how BCL2A1 may control p38 MAPK activation, we speculate that it may be related to its hypothetical capacity to directly or indirectly bind with or inhibit relevant intermediates required for the activation of this MAPK after TCR signaling. In this regard, BCL2A1 can be located in the cytosol of the cell and its expression is rapidly induced in CD4^+^ cells following TCR stimulation [19, 51]. This phenomenon, that has been initially proposed to be essential for the survival of CD4^+^ cells after their activation [51], may also play a role modulating their capacity to differentiate into potentially harmful pro-inflammatory T_H_17 cells during an autoimmune response. In addition to the regulation of p38 MAPK activation, BCL2A1 could modulate the differentiation of T_H_17 by other complementary mechanisms. In this regard, it can be mentioned that BCL2A1 interacts with the BH3-like protein Beclin-1, thus potentially contributing to the inhibition of autophagy [52] and that Beclin-1-deficient mice fail to mount autoreactive T-cell responses and are resistant to experimental autoimmune encephalomyelitis in association with a reduction in T_H_17 and T_H_1 cells [53]. Clearly, the characterization of the BCL2A1 interactome would help to elucidate the mechanism/s involved in the T_H_17 regulatory effect of BCL2A1 and to precisely identify this cell death regulator as a potential relevant target for the activation/differentiation of CD4^+^ cells and for the control of autoimmune diseases. Experiments are in progress to address these important questions.
